# Clinicians’ roles and necessary levels of understanding in the use of artificial intelligence: A qualitative interview study with German medical students

**DOI:** 10.1186/s12910-024-01109-w

**Published:** 2024-10-07

**Authors:** F. Funer, S. Tinnemeyer, W. Liedtke, S. Salloch

**Affiliations:** 1 Institute for Ethics, History and Philosophy of Medicine, Hannover Medical School (MHH), Carl-Neuberg-Str. 1, 30625 Hannover, Germany; 2 Institute for Ethics and History of Medicine, Eberhard Karls University Tübingen, Gartenstr. 47, 72074 Tübingen, Germany; 3 Faculty of Theology, University of Greifswald, Am Rubenowplatz 2/3, 17489 Greifswald, Germany

**Keywords:** Clinical decision support systems (CDSS), Artificial Intelligence (AI), Future health care professionals, Level of understanding, Explicability

## Abstract

**Background:**

Artificial intelligence-driven Clinical Decision Support Systems (AI-CDSS) are being increasingly introduced into various domains of health care for diagnostic, prognostic, therapeutic and other purposes. A significant part of the discourse on ethically appropriate conditions relate to the levels of understanding and explicability needed for ensuring responsible clinical decision-making when using AI-CDSS. Empirical evidence on stakeholders’ viewpoints on these issues is scarce so far. The present study complements the empirical-ethical body of research by, on the one hand, investigating the requirements for understanding and explicability in depth with regard to the rationale behind them. On the other hand, it surveys medical students at the end of their studies as stakeholders, of whom little data is available so far, but for whom AI-CDSS will be an important part of their medical practice.

**Methods:**

Fifteen semi-structured qualitative interviews (each lasting an average of 56 min) were conducted with German medical students to investigate their perspectives and attitudes on the use of AI-CDSS. The problem-centred interviews draw on two hypothetical case vignettes of AI-CDSS employed in nephrology and surgery. Interviewees’ perceptions and convictions of their own clinical role and responsibilities in dealing with AI-CDSS were elicited as well as viewpoints on explicability as well as the necessary level of understanding and competencies needed on the clinicians’ side. The qualitative data were analysed according to key principles of qualitative content analysis (Kuckartz).

**Results:**

In response to the central question about the necessary understanding of AI-CDSS tools and the emergence of their outputs as well as the reasons for the requirements placed on them, two types of argumentation could be differentiated inductively from the interviewees’ statements: the first type, *the clinician as a systemic trustee* (or “the one relying”), highlights that there needs to be empirical evidence and adequate approval processes that guarantee minimised harm and a clinical benefit from the employment of an AI-CDSS. Based on proof of these requirements, the use of an AI-CDSS would be appropriate, as according to “the one relying”, clinicians should choose those measures that statistically cause the least harm. The second type, *the clinician as an individual expert* (or “the one controlling”), sets higher prerequisites that go beyond ensuring empirical evidence and adequate approval processes. These higher prerequisites relate to the clinician’s necessary level of competence and understanding of how a specific AI-CDSS works and how to use it properly in order to evaluate its outputs and to mitigate potential risks for the individual patient. Both types are unified in their high esteem of evidence-based clinical practice and the need to communicate with the patient on the use of medical AI. However, the interviewees’ different conceptions of the clinician’s role and responsibilities cause them to have different requirements regarding the clinician’s understanding and explicability of an AI-CDSS beyond the proof of benefit.

**Conclusions:**

The study results highlight two different types among (future) clinicians regarding their view of the necessary levels of understanding and competence. These findings should inform the debate on appropriate training programmes and professional standards (e.g. clinical practice guidelines) that enable the safe and effective clinical employment of AI-CDSS in various clinical fields. While current approaches search for appropriate minimum requirements of the necessary understanding and competence, the differences between (future) clinicians in terms of their information and understanding needs described here can lead to more differentiated approaches to solutions.

## Background

Clinical Decision Support Systems (CDSS) are being increasingly introduced in various domains of health care for diagnostic, prognostic, therapeutic and other purposes. Clinical decision support as such has been discussed for decades [1], however, innovations in artificial intelligence (AI) and Machine Learning (ML) have intensified the debate on the chances and pitfalls of clinicians relying on computerised input. Potential benefits of the introduction of AI-CDSS in clinical workflows arise from the high accuracy of predictions in which AI-CDSS, even today, outperform human specialists in some tasks [2]. In addition, the efficiency of health care might be enhanced by employing computerised support, especially for simple or repetitive tasks that can be meaningfully entrusted to machines. On the other hand, risks are identified in such different fields as patient safety (e.g. alert fatigue), interoperability, user acceptance, users’ computer literacy, or disrupted and fragmented workflows [3]. Attempts to increase reliability and trustworthiness as well as technological harmonisation are considered to be key for the future success of AI-CDSS in health care.

From an ethical perspective, issues pertaining to the interpretation of established bioethical principles, such as justice or autonomy, concerning AI-CDSS have been discussed intensively in recent years. Regarding justice, for example, the right to equal access to health care plays a key role in enabling all patients to profit from newly introduced health care technologies as soon as their clinical benefit has been proven. More intricate ethical issues relate to testimonial (in-) justice if doctors are about to decide whether to trust a patient’s testimony or the outputs generated by a machine from big socio-demographic or clinical data [4]. Patient autonomy can be challenged in various ways by the introduction of AI-CDSS, for example, given the lack of clarity as to whether information needs to be provided about the use of an AI-CDSS in clinical care [5] and how much and what information needs to be provided to the patient to enable him or her to give informed consent on this basis. Additionally, the use of AI-CDSS can compromise the physician’s autonomy by making it difficult or even impossible for the clinician to assess recommendations, by unclear arrangements for integrating the AI-CDSS into shared decision-making, or by removing the clinician’s control over when and how the AI-CDSS is being used [6].

Other ethical issues relating to the introduction of AI-CDSS are closely linked to epistemological questions about the degree to which health care professionals are able to reproduce the outputs of computerised decision support. Some authors argue that “black-box medicine” “conflicts with core ideals of patient-centered medicine”, and it “is not conducive for supporting informed decision-making based on shared information, shared deliberation, and shared mind between practitioner and patient” [7]. The question of the need to understand an AI tool has become an important part of the discussion on the use of AI in health care, as it often seems to be closely linked to normative concepts such as trustworthiness, accountability and agency of health care professionals. A vivid discussion has emerged around the question whether “explicability” needs to be introduced as a further principle in the canon of bioethics when it comes to the evaluation of AI-driven tools [8, 9]. The term “explicability” is far from being unambiguous, and other terms, such as interpretability or transparency, are often used interchangeably [10]. The trade-offs that often need to be made between explicability and other goals in health care, such as accuracy, are also ethically meaningful [11]: the increased explicability in some applications comes at the cost of the accuracy of the predictions. In this respect, approaches to the necessary level of explicability of AI-CDSS are highly case-dependent and constrained not only by the clinicians’ skills and their own understanding but also by technical limitations that depend on the computational methods chosen in the development of medical AI [12].

Empirical research on stakeholder perceptions of the (ethical) chances and challenges of AI-CDSS are greatly needed to inform the debate and further guide technological development and policy-making. On the one hand, this can provide a reality check for the primarily conceptual-normative discourse and thereby test the validity of arguments in practice. On the other hand, explorative empirical research in particular can help to generate questions that may not have been asked so far. Initial qualitative empirical evidence exists on the perceptions, expectations and attitudes of clinical users of CDSS, as well as of barriers and facilitators of its use [13–16]. It generally appears that health care professionals working in hospitals are especially afraid of a loss of professional autonomy and the difficulties of integrating the systems into their clinical workflows [17]. Previous presentations of qualitative empirical results have succeeded in particular in collecting and mapping the breadth of ethical aspects that are considered relevant by health care professionals as a collective. However, greater differentiation is now required in order to achieve a greater depth of understanding of the underlying reasons for ethical aspects that are considered important.

Against the background of the current status of research, this article reports from a qualitative interview study exploring German medical students’ perceptions of ethical issues related to exemplary AI-CDSS. Medical students were selected as interviewees as they are future health care professionals who will most likely be dealing with AI-driven support throughout their professional careers. Due to their age their views on digital technologies might considerably differ compared to (senior) physicians who have been the participants of previous studies. Although, on the one hand, limited clinical experience can lead to different attitudes than those of experienced physicians, on the other hand, it can be stated that medical students at the end of their studies have initial insights and experiences in various clinical fields. They also have ideals and expectations regarding their own clinical role and responsibilities within health care. In this respect, we argue that medical students exhibit a characteristic that distinguishes them from experienced physicians, namely that their ideals of the professional role and associated responsibilities are not immediately relativised or compromised by everyday practical constraints. In this respect, the study presented here complements the empirical research with the views and attitudes of a further group of key protagonists for our future healthcare.

The qualitative interview study generated findings on various aspects of the use of AI-CDSS that have already been published elsewhere, such as on questions of the (final) responsibility of health care professionals [18] or on the necessity and scope of information and communication about the use of AI-CDSS [19]. However, a central theme of the interviews was the question of the clinician’s need for understanding and the competencies required to be able to use an AI-CDSS in a responsible manner. Here and in the following, “understanding” refers to how the interviewees consider it necessary to understand how a specific AI-CDSS works in general and how an individual recommendation come about. The need to “understand” the AI-CDSS and its recommendations in this way results in requirements for information about the AI-CDSS (cf. “explainability”/“explicability”) on the one hand and for the competencies required for its use on the part of the clinician on the other. The goal of the exploration was to understand the medical students’ attitudes and subsequently their reasons regarding the necessity of understanding to employ AI-CDSS. In contrast to previous studies, our approach enabled us not only to present the attitudes of future health care professionals but also to situate them within their epistemic-ethical context of justification, that means, to illustrate the connection of necessary understanding with the self-assigned clinical role as a doctor and its associated responsibilities. In this way, the different professional attitudes can be traced back to their premises and existing differences can be explained more comprehensively. On this basis, two types of medical students with different rationales can be differentiated. To the best of our knowledge, this study is the first to present such contrasting positions within the debate on understanding the requirements for medical AI in their context of justification elicited by qualitative research.

## Methods

A qualitative interview study was conducted to arrive at an in-depth understanding of medical students’ perceptions of ethical issues surrounding AI-CDSS with a special focus on the knowledge and competencies needed to use such systems in clinical practice. The interview guide and other key findings from the study, that also included nursing trainees and patients as interviewees, have already been reported elsewhere [18, 19]. Semi-structured interviews were conducted with advanced medical students at a German maximum-care hospital. Ethical approval was obtained from the local Research Ethics Committee prior to conducting the study (Reg. No. 9805_BO_K_2021).

### Data collection

Interview partners were included in the convenience sample if they met the following inclusion criteria: medical students in the fourth or fifth year of study, ≥ 18 years old and sufficient proficiency in German. There was no relationship established between the participants and the interviewer prior to the study; they met for the first time in the interview situation. Participants received some general information about the interview topic before the interview. Due to the COVID-19 pandemic, video calls were used for all interviews. They were conducted in German between June and July 2021. Most participants were at home and alone during the interviews. A common expense allowance was paid for participation.

The interview guide for the semi-structured interviews included two case vignettes that were presented during the interviews in written form and pictures (see “Medical Students’ Interview guide” within Supplement 1, published in [18]): The first vignette introduced an AI-CDSS to support doctors in the surgical setting (intra-abdominal surgical navigation) and the second presented an app for prognosis and therapy planning in chronic kidney disease. The AI-CDSS were selected with the aim of a variation in terms of the clinical field of application (surgery vs. nephrology), acute vs. long-term care and the degree of support (manual guidance, e.g. for incision lines, vs. prognosis estimation and therapy planning). The interviewees had the opportunity to discuss digitisation in health care in general, express their spontaneous reactions to the vignettes and then answered questions, for example, on patient information or competencies that must be expected from future clinicians. The interviews therefore have characteristics that combine both theory-generating expert interviews and problem-centered interviews [20]: The interview guide was structured on the basis of the debate in the literature, so that it addresses typical topics such as the question of the understanding of AI-CDSS, but is also open to the interview situation and the interviewees with regard to the scope and content discussed, thus allowing the interviewees to decide on the relevance and further exploration of the topic in the interview. The semi-structured interviews thus combine deductive and inductive methods. The interviews were audio-recorded and field notes were taken. We stopped conducting the interviews when saturation was reached, thus, at the point where additional interviews no longer generated new information relevant for the research question based on an iterative process of data collection and data analysis. Saturation refers to the characterization of the two argumentative types we identified in this study.

### Data analysis

Interviews were anonymised for people, places and institutions, and fully transcribed. The data analysis used key principles of qualitative content analysis according to Kuckartz [21]. Regarding this multistage procedure, inductive category building from the data is combined with theoretically derived categories that are defined prior to the start of the inductive analysis. In order to develop the deductive categories, topics related to the research question were extracted from the literature and subsequently interpreted in light of what emerged from the interviews. We documented coding rules for the deductive categories and selected exemplary passages (see Supplement 2, published in [18]). The data analysis was conducted by FF, ST and SS as researchers with interdisciplinary backgrounds in medical ethics, medicine, philosophy and pedagogics. MAXQDA (2020) was used as software to support the data analysis. The coding system was constantly revised and considerably expanded during the analysis. Ambiguities and disagreements which occurred were discussed critically between the authors and decided by consensus.

The interviews with 15 medical students (self-reported gender: 8 ♀ / 7 ♂; average age 25.5 years, range: 23–36 years) lasted an average of 55:49 min (with a range from 46:55 to 75:37 min). The interviewees had already finished all pre-clinical subjects, all clinical-theoretical subjects (e.g. pharmacology, pathology) and major clinical subjects such as surgery, internal medicine or emergency care. At this point in their studies, the students had been in full-time practice in hospitals for at least five months.

The results presented in this article are drawn from the overarching categories “Reliability of the technology”, “Traceability/Comprehensibility of decisions” and “Competencies” (see Supplement 2, published in [18]). The reporting of methods and results was guided by the Consolidated Criteria for Reporting Qualitative Research [22]. Exemplary passages supporting the main findings were translated from German to English by the authors to be included in this article. Each of the interviews was analysed in its overall epistemic-ethical context of justification and explanation of premises. This made it possible to differentiate between different types of attitudes and their justifications among the future clinicians.

The focus of the results reported in this article lies on self-perceived clinical roles and necessary levels of understanding when using AI-CDSS. Different patterns of justification for the interviewees’ convictions regarding these topics were identified. Based on the interviewees’ statements, we have inductively reconstructed two major types to illustrate the most important alternative justification patterns: on the one hand, *the clinician as a systemic trustee* (“the one relying”) and, on the other hand, *the clinician as an individual expert* (“the one controlling”).

We first introduce the common starting points of these two argumentative types in the results section. Subsequently, the two types are reconstructed and their different patterns of justification are elaborated in parallel using interviewees’ statements. A tabular overview then compiles the key characteristics and elements of the alternative justification patterns (see Table 1). From this, we illustrate argumentative challenges that emerged in some interviews and with which the interviewees said they would be confronted in dealing with medical AI decision support.

## Results

The interviewees’ statements represent requirements they placed primarily on themselves but claimed them to be generalisable to “clinicians” or “doctors” (cf. e.g. SI-5, SI-11). While some of the interviewees could be assigned quite easily to one of the two argumentative types^1^, there were some interviews in which parts of both reasoning patterns were combined. There is also a continuum between the two types, with individuals who can sometimes be more attributed to one type, and sometimes more to the other.

### Starting point of both types: scientific proof of benefit

Both argumentative types indicate that they need scientific evidence on the clinical validity for the use of AI-CDSS outcomes. Specifically, interviewees believe that a positive effect of using AI-CDSS compared to not using it must first be demonstrated. In this respect, clinical decisions made by clinicians with an AI-CDSS should be proven to be correct at least significantly more frequently than comparable decisions made by average specialists without an AI-CDSS (cf. e.g. interviews SI-1, SI-4, SI-6, SI-7, SI-13 and SI-14):

*And*,* yeah*,* otherwise maybe like validation studies*,* to what extent the things that the device predicted or recommended were actually good compared to more traditional methods or something like that.* (Stud_Interview_10, Position: 70)

Other criteria which would have to be evaluated, one interviewee said, are benefits, such as the following:

[T]hat would be, for example, fewer complication rates after surgery, shorter surgery duration, in other words, all kinds of things that would be beneficial to the patient. And, of course, also for the surgeon. (Stud_Interview_12, Position: 29)

In addition, regular re-evaluations should detect long-term changes in human-machine interaction and make them assessable regarding their outcomes (cf. SI-4, SI-6 and SI-7):

There has to be a superiority that if you work with this support system now, that it really brings the advantages that you expect. […] But you can really only find that out over time by comparing it with each other, whether it really brings advantages and fewer complications occur, for example, and the duration of surgery is shortened and so on. (Stud_Interview_7, Position: 33–35)

According to some of the interviewees, the existing evidence on AI-CDSS should be reviewed, assessed and approved by appropriate expert bodies, such as governmental authorities or medical societies in the relevant field (cf. e.g. SI-6), before clinical deployment.

Based on this common starting point of sufficient evidence and suitable instances for evaluating it, the interviews reveal considerably different positions on how clinicians should deal with the scientific evidence of a positive proof of benefit. Two main argumentative types will now be reconstructed.

### Reconstruction Type I (“the one relying”)

Regarding “the one relying”, errors and causing harm are an inevitable part of medical practice (cf. e.g. SI-3, SI-6 and SI-12):

You simply have to say goodbye to that [ = the idea of not making mistakes; authors]. There are always mistakes somewhere and hopefully they will be less with this programme, but mistakes and misjudgements do happen. (Stud_Interview_12, Position: 85)

Empirical evidence of better outcomes and lower error rates with the help of AI-CDSS is, therefore, decisive for the clinician’s decision on the use of this technology:

[…] that I personally, as soon as it was empirically shown that this surgical assistant works well and brings better results, that I would trust it very well, probably also more than people who operate without this assistance. (Stud_Interview_6, Position: 47)

It’s just a question of who is better or who makes fewer mistakes, whether you can rely on it more or not. (Stud_Interview_14, Position: 41)

Even in the case that damages were caused associated with the use of an AI-CDSS, retrospectively, the use could have been better justified than the non-use:

But, nevertheless, it would have been the most rational thing to do, even if the end result is a worse outcome. In my view, it would still have been the most rational thing to do, or the most appropriate thing to do. Basically, to consider that it is more likely that this outcome will not occur. (Stud_Interview_6, Position: 115)

The goal of “the one relying” is, thus, to cause as little harm as possible. Protagonists of this type see it as necessary that it has been empirically shown that a higher benefit can be achieved with AI-CDSS’s help. In this respect, the use of AI-CDSS is understood to be the evidence-based best available remedy to achieve the desired benefit for most cases. Accordingly, the clinician is also not responsible for damages resulting from the AI-CDSS recommendation because, based on the empirical evidence, its use was indicated (cf. e.g. SI-1 and SI-6). The prerequisite, however, is that the clinician correctly informed the patient about the potential damages beforehand (cf. e.g. SI-3).

Regarding “the one relying”, this position is reflected in a necessary level of understanding that essentially consists of two components: knowing that appropriate regulatory authorities and processes exist that have verified the scientific evidence of benefit, for example, as the result of a certification process or a recommendation by medical societies (cf. e.g. SI-3 and SI-6). This knowledge of appropriate processes is also framed as trust in the existing system:

[…] but at a certain point, there’s just a certain amount of trust that’s necessary, and I just have that trust in the people who programmed this system. (Stud_Interview_6, Position: 153)

However, an understanding by the clinician of how the AI-CDSS works and how it arrives at its outcomes is held to be unnecessary by “the one relying”:

I didn’t mean that I have to understand the system. I don’t really have the […] major interest in that. So, as long as I’m told that it’s been empirically shown that this system works, I’m not so incredibly interested in how this system comes to the benefit, if I’m honest. (Stud_Interview_6, Position: 71–73)

According to this understanding of the clinician’s role in dealing with an AI-CDSS, there is a responsibility of the clinician to ensure that information about advantages and disadvantages or risks of its use is correctly conveyed to the patient (cf. e.g. SI-1 and SI-3). The evidence that decisions with AI support are generally better than without it justifies the acceptance of potential errors that are caused by the AI-CDSS or occuring during its use:

But I still think that if it was really shown that mine [ = my decision; authors] is usually worse than the AI’s and then I end up accepting fewer mistakes and preventing many mistakes on my part in return, then it was still the right decision to follow. In my opinion, it would be a bad decision not to trust the AI just because it might sometimes make different mistakes than I do. (Stud_Interview_6, Position: 85)

### Reconstruction Type II (“the one controlling”)

The other argumentative type identified from the interviews shows, in some respects, a similar argumentative pattern as the first one; in other respects, major differences emerge. Similar to the first type, “the one controlling” acknowledges the occurrence of errors and harm in the context of medical practice (cf. e.g. SI-2, SI-5 and SI-12). They also aim for the lowest possible number of errors and damage. However, the task of avoiding harm is seen as being anchored individually in the role of the clinician: “the one controlling” tries to compensate or reduce sources of error for the individual patient as best as possible (cf. e.g. SI-5 and SI-8). Therefore, the clinician is in the role of always questioning the outcome of an AI-CDSS and judging whether the outcome is correct for the patient’s unique situation (cf. e.g. SI-1, SI-2, SI-5, SI-7, SI-8, SI-9, SI-10 and SI-13):

Then, of course, the doctor really has to check whether this app or, yes, this support has then also decided correctly for him, so to speak. (Stud_Interview_2, Position: 87)

The clinician is in the position to consider the context, the neglected aspects or the entirety of the patient’s situation more comprehensively than the AI-CDSS ever can (cf. e.g. SI-4 and SI-9):

And that’s also interesting, for example, […] sometimes things are a bit trickier than you can type them in [ = in the input data set of the AI; authors], I’d say, when someone describes them to you. (Stud_Interview_4, Position: 59)

[A]s a clinician, you could almost just rely on all sorts of computer systems and then you wouldn’t need people at all. […] But I think it always needs that one person who can somehow connect everything together a bit and who then also takes responsibility for interpreting something out of it. (Stud_Interview_9, Position: 29)

If, despite the critical dealing with AI-driven recommendations, errors occur because the clinician has inadequately checked its outcome, according to “the one controlling”, it is the clinician who has failed:

And, accordingly, that is then ultimately medical malpractice, if he then blindly trusts the machine. (Stud_Interview_12, Position: 37)

In this respect, the recommendation of an AI-CDSS is only another element that can assist in identifying a correct decision, but its recommendation must be evaluated in the context of clinical guidelines, empirical data and consensus. Basing a decision solely on the information provided by an AI-CDSS does not constitute sufficient justification:

We always have to justify what we do. And we do so on the basis of guidelines that rest on data, facts and consensus. And if this app plays a role, then that’s part of it. If I relied on the app only without checking the scientific basis for it, then it’s my fault. (Stud_Interview_13, Position: 87)

In summary: “The one controlling” argues that harm is to be reduced and it is good if this goal is improved by AI-CDSS in an evidence-based way. However, the clinician has to complementarily consider the limitations of the AI-CDSS and prevent potential harms that may be caused by its use. According to “the one controlling,” the clinician is not only in the role but has the responsibility to control and judge whether the AI-CDSS’s recommendation is appropriate for the case at hand (cf. e.g. SI-2, SI-4 and SI-9):

I would never say that the system should be allowed to take the decision away from me, honestly. So, I think the system can support me in that, yes, but ultimately, I still have the responsibility. (Stud_Interview_2, Position: 109)

A sufficient level of understanding is required to enable the clinician to consider the system’s limitations (cf. e.g. SI-2, SI-5, SI-11 and SI-14):

If you don’t understand that [ = how the CDSS comes from its input to its output; authors] or you don’t understand the basic idea behind it, I would be afraid that you’re relying way too much on systems like that way too quickly. And if you don’t understand what’s happening in the meantime, what’s happening inside the device or inside the system, I would also think that you yourself can’t control what comes out of it anymore. And if you use a system like that, I think you should also control yourself what’s happening and not rely on it blindly. (Stud_Interview_11, Position: 35)

“The one controlling” knows about his/her own limits of understanding because of his/her own qualification in medicine (cf. e.g. SI-2 and SI-5) but demands at least enough understanding to be able to use it competently in the context of his/her own medical practice:

So that I can use it optimally, honestly. Because, of course, I’m not a physicist and not a mathematician. […] But I should definitely have a basic knowledge of how this comes about. (Stud_Interview_2, Position: 111)

Some interviewees of this second type consider it necessary to know the advantages and disadvantages and specific risks of AI-CDSS use (cf. e.g. SI-2 and SI-10). They desire to know about the regulatory review procedures and certifications by experts (cf. e.g. 8) and to have a basic knowledge of how ML and neural networks function (cf. e.g. 2, 5, 8), and how the system arrives at a specific recommendation (cf. e.g. SI-2, SI-5, SI-8 and SI-9). They also want to know about the data basis and the origin and context of the data (cf. e.g. SI-5, SI-7, SI-8, SI-10 and SI-13). Sufficient clinical experience of the clinician regarding the treatment, before using an AI-CDSS, is also seen as necessary to adequately assess the quality of a recommendation (cf. e.g. SI-7).

That means, from my point of view, either the basic data collection or the way to get there would have to be somehow transparent, that I as an end user of this AI can somehow assure myself that this algorithm has also drawn the right conclusions from right data and not from wrong data the 99% right conclusions and at 1% it always comes back to the error and I rely 100% on this AI, though. (Stud_Interview_8, Position: 31)

And I think there should be a certain transparency in it or a certain explanation. So, if I can’t understand how this support system comes to this cut or to this position, then I would have to be able to understand, okay, how do you analyse the other structures around it that you come to the conclusion that that’s exactly where the cut should be. (Stud_Interview_8, Position: 25)

Attaining such a level of understanding requires, on the one hand, the competencies mentioned on the part of the professionals and, on the other hand, an appropriate presentation of the information by the AI-CDSS:

So, of course, I would prefer to inquire, […] so, in the best case, the system could somehow explain to me how it came to this decision, so I know that it first explains or first marks which structures it has recognised and then next makes the cut, so that I can just reassure myself: “aha, maybe the programme has recognised a structure incorrectly and has come to a wrong cut.” Then I could follow up on this error and say, okay, there’s a mistake here, that’s why I don’t take over this cutting direction. (Stud_Interview_8, Position: 45)

The understanding is particularly relevant to inform patients adequately (cf. e.g. SI-2 and SI-8) and be empowered as a clinician to “intervene” during the use of the AI-CDSS when needed (cf. e.g. SI-5 and SI-13):

I know what features there are, but I also know how to turn those off and I know my fallback level. How much the system can interfere with me, I’d say, and then how I could bypass that. (Stud_Interview_13, Position: 41)

Only a comprehensive understanding would allow the clinician an informed assessment of the system’s limitations and prevent an overestimation of its performance:

[t]o make sure that you don’t hopelessly overestimate it. It’s not like some God-given thing that suddenly knows everything. It also has its limits, and one should be clear about that. (Stud_Interview_10, Position: 116)

**Table 1:** Synopsis of the characteristics of the two argumentative types

	Clinician as systemic trustee (“the one relying”)	Clinician as individual expert (“the one controlling”)
Clinical validity of the AI-CDSS (i.e. scientific evaluation)	positive proof of benefit as a necessary precondition	positive proof of benefit as a necessary precondition
Attitude towards error rates or potential harm	errors are part of medicine with and without the use of AI-CDSS	errors are part of medicine, but the clinician needs to mitigate these errors for individual patients as well as possible
Role of the clinician	user of an application that statistically causes the least harm	user of an application whose errors must be controlled by him/her critical questioning and review of AI-CDSS outputs to avoid errors consideration of each patient’s context and potentially neglected factors
Accountability for harm caused by AI-CDSS	The clinician bears no accountability because AI-CDSS’s use was evidence-based indicated. The patient needs to be informed about potential harms beforehand.	Overreliance on AI-CDSS recommendations is culpable, leaving clinicians accountable for some errors that should have been identified previously. Final responsibility for treatment remains with the clinician.
Goals and ways to achieve it	minimising harm by using AI-CDSS outcomes	minimising harm and the potential harm arising from AI-CDSS outputs through clinician review
Necessary level of understanding	sufficient understanding to deal with the patient’s information needs, i.e. − knowledge of the advantages and disadvantages/risks − knowledge of a regulatory process for reviewing clinical validity	sufficient understanding to evaluate AI-CDSS outputs as well as deal with the patient’s information needs, i.e. − knowledge of the advantages and disadvantages/risks − knowledge of a regulatory process for reviewing clinical validity − basic information technology knowledge (“How does machine learning work?”) − knowledge about underlying dataset and its limitations − understanding the conclusion of a recommendation (“How does the AI-CDSS arrive at this outcome?”) − in some cases, enough previous clinical experience, being able to make supported decisions even without AI-CDSS

## Discussion

Expectations and requirements for the design of human-AI collaborations in health care contexts have been in the focus of philosophical and ethical publications for a few years now [3, 6, 23–28]. Particularly questions about the epistemological quality and limitations of AI-generated recommendations and the resulting ethical questions about the morally legitimate way of dealing with these chances and limitations have attracted attention. The question of whether a highly reliable or accurate AI recommendation is sufficient, or whether and to what extent it must be explainable to justify a diagnostic or treatment decision based on it from an epistemic and ethical point of view was often at the core of the analyses [7, 10, 29–31]. This is discussed mostly in the context of a potential loss or diffusion of responsibility and accountability [32–38]. Our results show that this complex question also affects the interviewees and they see it relevant for their own future clinical practice and, for instance, whether alternative subjects of responsibility could be assigned [18]. Many of the arguments found in the literature could also be found similarly or even the identically among the interviewees.

All interviewees consider themselves as representatives of evidence-based medicine. Scientific proof of benefit (or clinical validation) was seen as the most important starting point for the use of applications such as AI-CDSS in health care (cf. similarly [14]). The interviewees only considered the use of AI-CDSS worthy of discussion if it was proven that it achieves at least a comparably good performance and outcome as clinicians achieve without AI-CDSS (cf. also [16]). The more reliable the evidence, the more obvious or even imperative the use of the application would be. The rationale for this imperative is the recognised goal of medical practice, to maximise patient benefits, or, more precisely, to serve the well-being and will of the patient (cf. [6]). From the evidence-based positive proof of a benefit for patients, therefore, follows the necessity to pursue the potential of AI-CDSS, wherever this is feasible (cf. [10]).

Decisively, however, the argumentative justification for one or another answer to the question about the proper way to deal with this scientific proof of benefit is decided by the image of the clinician’s role the interviewees have. From this “professional role”, they derive, accordingly, which tasks they have to fulfil, which accountability for the clinical decision-making process this entails, and which competencies they need to guarantee this accountability – or, in other words, which moral obligations go hand in hand with it.

The students interviewed anticipate their future role as clinicians as to have the moral obligation, in the context of the respective health system, of selecting and suggesting to patients those diagnostic and treatment options that cause the least harm and the most benefit, based on evidence. However, while the interviewees of the type “the one relying” recognise this goal as being best pursued by using an evidence-based AI-CDSS to statistically benefit the most patients, which is held to be based on a broad database and trained neither to underfit nor overfit, the interviewees of the type “the one controlling” add to this requirement the need to check the validity of the specific recommendation of the AI-CDSS for the individual patient in the given situation. Thus, while some rely on the evidence-based validity of the positive proof of benefit for AI-CDSS in a collective and consider that to be sufficient to identify the greatest possible patient benefit, others focus on nonetheless possible limitations of AI-CDSS statements that may limit their evidence-based validity for the individual patient (even if statistically such an approach might result in more frequent errors, cf. [10]).

This is a well-known epistemic-ethical conflict about how to achieve the greatest possible benefit: either by striving for the greatest possible benefit for the entire group (and thus indirectly for each individual on average) or directly by striving the greatest possible benefit for the individual patient. This trade-off is not specific for AI applications. Instead, it is rather a generic problem of applying generally functional measures or tools with existing limitations to individual cases. However, this challenge is made all the more apparent by the knowledge about limitations and biases of data and AI applications built on them (cf. [15], also for doubts about the robustness of data). The use of AI would be most widely accepted [15, 17] and unobjectionable from an ethical point of view if its users could be sure that the AI-CDSS could not make any mistakes. However, there will probably never be error-free datasets (e.g. due to noise or recording errors and biases) [10], which always implies false-positive and false-negative AI-CDSS predictions. This means that compromises will always have to be made. The following ethical question, thus, arises concerning what is the minimum quality of the data and how should recommendations from them be handled in view of their limitations in order to make decisions about the quality and length of life for an individual patient. As Amann et al. argue, in the context of AI use, the principle of non-maleficence urges clinicians not to harm their patients “either intentionally or through excessive or inappropriate use of medical means” [10] and, furthermore: “This is why, from a medical point-of-view, not only clinical validation but also explainability plays an instrumental role in the clinical setting” [10]. Similarly, the obligation to benefit and not to harm the individual patient urges future clinicians of the type “the one controlling” to avoid, if possible, patient injury due to inappropriate care – this could only be achieved for them through sufficient scrutiny of the appropriateness of the decision in question (cf. [10, 16]). Accordingly, our interview results shed light on the debate on the importance of explicability in medical AI and trade-offs that sometimes need to be made with other goals in healthcare. According to the point of view of “the one controlling”, explicability might rather serve as a means that is important to prevent patients being harmed by the use of medical AI. In general, this argumentative type strives for a sufficient understanding of AI-CDSS and its outputs to provide optimal care as well as to deal with the patient’s information needs. “The one relying” as the second argumentative type we identified also upholds minimising harm when using AI-CDSS but does not strive for understanding of the machine outputs to the same degree as the other type does. While interviewees of the one argumentation type call for explicability, under the assumption that this will allow them to better prevent harm to individual patients and to better inform them (and thus better benefit the patient), interviewees of the other argumentation type call for less explicability, under the assumption that this will statistically allow more decisions to be made that benefit the patient. Hence, our results might enrich the – so far predominantly theoretical – debate on explicability of medical AI in highlighting and discussing different needs as perceived by future healthcare professionals.

Another aspect, which was raised especially by interviewees of the type “the one controlling”, concerns the inadequacy of AI-CDSS to consider only those factors that can be operationalised. Clinicians would have to take into account these aspects which are associated with the patient’s personality, values, life situation and socio-cultural background (cf. similarly [29, 39]), as these realised relevant aspects of patient autonomy. This aspect is not addressed by interviewees of the type “the one relying”; whether due to the fact that they consider these aspects to be operationalisable, or if they consider them to be of secondary importance, cannot be said on the basis of the data. However, this aspect seems all the more necessary the more routinised the use of AI-CDSS in clinical practice becomes [39] in order to continue to meet the needs, wishes and preferences of individual patients in the future.

It can be summarised that the future clinicians interviewed read the evidence about the positive proof of benefit with existing limitations against the background of their respective conception of the role of the clinician and its moral obligations.

Correspondingly, the accountability or responsibility for harm prevention is also considered to be realized in different ways when AI-CDSS recommendations are passed on: for some through the evidence-based, indicated use of the AI-CDSS and transparent information about their limitations, for others only through the critical review and the validation of the respective AI-CDSS recommendation regarding the individual case. There is agreement among the interviewees, but also in other empirical studies [14, 15, 18], about the importance of the clinician’s responsibility when using AI support; however, the ways in which this responsibility can be executed varies greatly among the interviewees, as our results show.

According to the interviewees of the type “the one relying”, in order to fulfil the role and moral obligations in dealing with AI-CDSS, clinicians need a sufficient understanding of the advantages and disadvantages and existing risks of the use of a certain AI-CDSS to be able to communicate them to patients for the latter’s informed consent. Knowledge of rigorous validation processes for assessing the evidence of benefit and regulatory standards, for example, through government authorities, medical societies and/or certification according to medical device regulations, is sufficient reassurance for them to use an AI-CDSS (cf. similarly [16, 10]).

By contrast, interviewees of the “the one controlling” type demand a more comprehensive understanding from clinicians that enables them to critically review and interpret AI-CDSS, their single recommendations and underlying assumptions (cf. [40]). Clinical decision-making has been carried out so far by clinical experts primarily on the basis of medical reasons [29, 35] and not only based on data. Explanations enable clinicians to interpret AI-driven recommendations in light of the respective situation and the individual patient [29] and to align them with their own medical clinical judgment [7]. Both the interviewees and the literature concede that different levels of understanding and explanation need to be achieved for different decision-making scenarios in everyday clinical practice, depending on the different risks and impacts on the patient’s life [10, 30]. More extensive competencies are required to fulfil this kind of clinician role, and higher demands need to be placed on the explicability of the AI-CDSS itself. Not being able to fulfil their role and meet the moral obligations is seen as a normative barrier to the use of AI-CDSS by future clinicians of the “the one controlling” type (cf. similarly the “distancing” of clinicians when the rationale for an AI-CDSS recommendation could no longer be understood: 14).

The need for competencies and knowledge as expressed by professionals is already known [10, 15, 16, 18], which is why the discussion on tailored training and professional development regarding the use of AI in clinical practice has recently gained momentum. Initial consensus studies are attempting to identify the skills and learning objectives that clinicians need to use AI tools (see, e.g., [41]), and national and international initiatives to integrate such into curricular structures have been launched; however, there is still often a lack of standardized training and study programs that would be available everywhere (cf. [42]). In a study at two German medical schools, it has also been shown that there is a positive correlation between AI literacy and students’ positive attitudes towards AI (cf. [43]). Our study adds to this existing knowledge in differentiating between two types of students that differ in their demand for education and competencies in so far as they perceive different levels of understanding to be necessary for using AI-CDSS in practice. Both types, however, share the view that future clinicians must be equipped with the appropriate skills to be able to meet the normative demands that stem from their professional role. This includes, for example, knowledge about the advantages and disadvantages/risks of specific AI-CDSS, regulatory processes for reviewing the clinical validity, basics of information technology and competencies to assess the underlying dataset and its limitations, and the reasonableness of a recommendation. Approaches, such as that proposed by Sand et al. [36], based on “Entrustable Professional Activities” appear to be particularly constructive for this purpose. With the help of such frameworks, necessary competencies can be identified in order to be able to ascribe certain responsibilities. For both cases, it will be possible to say: “Being a competent operator of such systems […] demands more from physicians than becoming information specialists. It requires a more general awareness of the fallibility of these systems and the various ways in which their utilization might fail” [36]. The answer to the question about the appropriate scope of competencies of clinicians will, nevertheless, be measured by the extent to which they should be able to safeguard control over the clinical decision-making process: clinicians of “the one relying” type will be able to get by with significantly fewer competencies than those of “the one controlling” type. However, this is only due to the different assigned role expectations and responsibilities of clinicians.

The results of our study underline that the reference to proof of high accuracy and the need explicability or understanding are by no means contradictory. In so far, our study adds an empirical perspective to the debates on explainable AI that have a predominantly technical or theoretical character so far. While some of the future clinicians interviewed can be linked to one argumentative type, others can be categorised as belonging to the other type; however, only a small number of those interviewed use both references to realise their conception of the clinical role along with its moral obligations. For the future clinicians, both represent approaches from which they deal hermeneutically with existing theory and evidence in order to be able to best fulfil their idea of the clinician’s role and its responsibilities – in each case, with the goal of serving the well-being and will of the patient. As clear as the normative preference for AI-CDSS use may be (if scientific proof of the benefit is provided), it becomes clear that the epistemological requirements for ensuring the benefit pledged for the individual patient follow different rationales. The future clinicians interviewed assess the trade-off between a normatively imperative maximum benefit and an (also normatively imperative) epistemic certainty to achieve this benefit differently (cf. similarly the conceptual analysis in [31]).

Limitations that need to be considered in the interpretation of this study’s results arise from the sample and the recruitment process. The study mirrors the perceptions and attitudes of German medical students from one university and cannot be generalised unconditionally. It could be that even more argumentative types were identified when drawing on a different (and broader) sample of study participants. Furthermore, each interviewee’s clinical experience is very limited so far, and they have minimal or no personal experience in dealing with AI-CDSS in clinical practice. Their answers therefore have a hypothetical character, insofar as they could act differently and formulate different claims in practice than in the interview situation based on the case vignettes. Although this limitation must be taken into account in the interpretation, we believe that the limited practical experience also has the advantage that positions are developed based on personal convictions and are not relativised too quickly against the background of practical feasibility. However, clinically experienced practitioners could possibly contribute to the identification of further argumentative types. Finally, the study results as reported in this article do not represent an encompassing analysis of AI-CDSS but are limited to certain aspects related to necessary levels of understanding as perceived by the stakeholders. It should therefore not be wrongly assumed from the results that other aspects were irrelevant for the interviewees.

## Conclusions

The ethical debate on the employment of AI-CDSS and its impact on physicians’ practice and professional role is already in full swing. Empirical evidence on the stakeholders’ own viewpoints, however, is limited so far. This study generated insights into prospective German clinicians’ perspectives regarding their professional role and necessary levels of understanding and explicability needed as a basis for responsible clinical decision-making. Two contrasting types of clinicians were particularly identified who differ, for example, in their perception of which level of understanding they perceive as necessary for AI-supported clinical decision-making.

The study results open up the debate on the levels of competencies needed and appropriate training programmes and professional standards (e.g. clinical practice guidelines) that enable the safe and effective clinical employment of AI-CDSS in various clinical fields. Future initiatives in this direction need to be aware that clinicians are not a homogenous group at all, either in their AI-related competencies or in their appreciation of which levels of understanding and explicability they consider necessary to undergird their professional judgment. Consensus-seeking processes, thus, might be necessary in the medical profession to ensure consistent standards which will enhance the trustworthiness of AI-supported health care.

From a research perspective, our hypothesis-generating study could be taken as groundwork for a more in-depth or quantitative exploration of the different types of professional users of AI-CDSS. In addition, more empirical studies in various national contexts are needed because expectations towards technological progress and the understanding of human-machine interaction differ greatly depending on the cultural context. Such research should not only elicit health care professionals’ perspectives but also generate evidence on patients’ viewpoints on the levels of explicability and transparency needed when AI is integrated in clinical decision-making. It is generally desirable that open and informed communication about the use of medical AI finds its place in patient-physician communication and shared decision-making so that patients’ information needs and treatment preferences can be adequately addressed. As a prerequisite, however, more work is needed to enhance the explicability of AI-CDSS (e.g. through visualisation) and increase physicians’ competencies in dealing with medical AI.

## Data Availability

The datasets generated and/or analysed during the current study are not publicly available as they might contain information that could compromise research participant privacy and consent.

## Footnotes

1 It was possible, for example, to assign interviewees No. 3 and 6 quite clearly to Type I and interviewees No. 2, 5, 7, 8, 9 and 10 to Type II.

## Abbreviations

AI: Artificial Intelligence
CDSS: Clinical Decision Support Systems
ML: Machine Learning
